# The Impact of Choir Singing on International Students’ Sense of Belonging, Loneliness, and Wellbeing: A Controlled Evaluation of UQ Voices

**DOI:** 10.3390/bs15050575

**Published:** 2025-04-24

**Authors:** Rong Han, Vicki Bos, Fiona Wiebusch, Mary C. Broughton, Genevieve A. Dingle

**Affiliations:** 1 School of Psychology, The University of Queensland, St Lucia, QLD 4072, Australia; rong.han@uq.net.au (R.H.); vickibos@teachinginharmony.com (V.B.); 2 The Queensland Institute, Brisbane, QLD 4067, Australia; fiona@thequeenslandinstitute.com; 3 School of Music, The University of Queensland, St Lucia, QLD 4072, Australia; m.broughton@uq.edu.au

**Keywords:** international university students, choir singing, sense of belonging, loneliness, wellbeing, community

## Abstract

International students beginning university face challenges, including loneliness and isolation. Research shows that choir singing can enhance social bonds and emotional wellbeing. However, its effect on international students remains underexplored. This study applied a social identity perspective to evaluate the impact of participation in a university community choir on international students’ university identification (i.e., sense of belonging), loneliness, and wellbeing. A non-randomised controlled study was conducted with 53 international students at UQ, including 31 UQ Voices choir members (*M*_age_ = 24.90, 71.0% female) and 22 non-choir students (*M*_age_ = 24.18, 72.7% female). Participants completed measures of university identification, loneliness, and wellbeing at baseline (pre) and after 6–8 weeks (post), along with group-based psychosocial resources measures. Data were analysed using 2 (choir, control) × 2 (pre, post) ANOVAs. A significant interaction effect emerged for university identification (sense of belonging), with choir participants improving more than controls. A main effect of group emerged for wellbeing, with choir members reporting higher wellbeing. No significant effects for loneliness, which was not elevated at baseline. Choir identification was significantly related to psychosocial resources (self-esteem, control, meaning and purpose, and mood improvement). Choir singing may serve as a preventative intervention to support international students’ wellbeing by fostering a stronger sense of belonging in the new university and country.

## 1. Introduction

### 1.1. International Students’ Experiences

Although all new students may find the transition to university challenging, international students often face additional challenges. These may include cultural adjustment, language barriers, and separation from established support networks (Alshammari et al., 2023; Dingle et al., 2024; Kristiana et al., 2022). As a result of these challenges, loneliness and a lack of a sense of belonging to the university are common among international students, affecting around two thirds of international students in various studies (Diehl et al., 2018; Dingle et al., 2022). Loneliness was further exacerbated during the COVID-19 pandemic, when international students faced extended travel restrictions, social isolation, loss of campus community, and exclusion from local support networks (Ellard et al., 2023; Nguyen & Balakrishnan, 2020). Indeed, first-year international students in Australia experienced higher levels of loneliness, greater psychological distress, and lower wellbeing during the first year of the pandemic compared to the year before the pandemic (Dingle et al., 2022, 2024). Furthermore, research has shown that loneliness negatively affects international students’ mental health and wellbeing, including an increased risk of depression and anxiety (Girmay & Singh, 2019). It can also increase feelings of alienation and make it harder for students to integrate into the university community (Wawera & McCamley, 2020).

These trends highlight the importance of cultivating students’ sense of belonging within the university. Belongingness involves feeling accepted, valued, and connected to a group or community, supported by strong social networks and meaningful relationships within the university (Glass & Westmont, 2014). Research has shown that the sense of belonging in higher education plays an important role in promoting both psychological wellbeing and academic achievement (Allen et al., 2024; Crawford et al., 2024). For example, a study of 3837 international students in Germany found that a strong sense of belonging predicted better wellbeing, higher study satisfaction, and a lower likelihood of dropping out (Yildirim et al., 2021). Similarly, a study of Asian international students found that a strong sense of belonging on campus reduced psychological distress, supported cross-cultural adjustment, and enhanced academic engagement (Self & Swank, 2023). Although a sense of belonging has significant positive impacts, international students often report feeling less welcome and respected on campus compared to their peers (Van Horne et al., 2018). Hence, these findings emphasise the need for targeted efforts to reduce loneliness and enhance the sense of belonging among international students.

### 1.2. A Social Identity Perspective

This study was informed by a social identity theoretical framework which suggests that group memberships affect our wellbeing when they contribute to our self-concept (Tajfel et al., 1979; Turner et al., 1994). Also known as the ‘social cure’ framework, the social identity theory applied to health and wellbeing (C. Haslam et al., 2018; Jetten et al., 2012) explains that shared social identities (such as ‘us St Joseph’s College students’ or ‘us members of team X’) provide important psychosocial resources to group members (Steffens et al., 2016). These resources include self-esteem, belonging, and social support (Greenaway et al., 2015; Jetten et al., 2014). According to this social cure framework, loneliness occurs when people experience a lack or loss of social group identities and associated psychological resources (S. A. Haslam et al., 2024; Hayes et al., 2022). Loss and lack of groups and associated identities often happens during life transitions such as moving to university (Griffin et al., 2025; Praharso et al., 2017). It follows, then, that efforts to maintain existing group memberships (Cruwys et al., 2020) and to join new groups with which students identify can provide these important psychological resources and promote students’ adjustment and wellbeing over time at the new university.

Choir singing is increasingly recognised as a beneficial activity to promote social bonds and improve wellbeing, offering young people a unique opportunity to build a sense of community and belonging through shared musical experiences (Bailey, 2021; Clift & Hancox, 2001; Glew et al., 2021; Van Der Sandt & Nardi, 2024). Conceptually, choir singing involves individual contributions to achieve group goals (e.g., synchronised movements and harmonised sound), which offers participants a shared experience that is likely to enhance the development of an additional social or choir group identity. In fact, the social cure theory has been validated in social prescribing programmes that include group singing and other community social activities such as creative writing (Evers et al., 2024; Hayes et al., 2024) and in community-dwelling adults involved in singing, instrumental, and dance groups (Draper & Dingle, 2021; Hendry et al., 2022). It has been validated in relation to group singing in older adults (Dingle et al., 2020; Howlin et al., 2022), in adults experiencing health conditions such as post-stroke aphasia, Parkinson’s disease and dementia (Forbes, 2021; Tamplin et al., 2024; Tarrant et al., 2021) and in adults experiencing chronic mental health conditions such as bipolar disorders and recurring depression (Dingle et al., 2013; Williams et al., 2019, 2020).

Together, these studies have established that psychological resources found to flow from social identification with a choir group include cognitive health, social connectedness, belonging, self-esteem, and mental wellbeing. However, social cure theorising has not yet been applied widely in studies of adolescents and young adults (Finn et al., 2023), and to our knowledge, it has not been applied to international students joining a choir at university. Therefore, questions remain about how choir singing might help these students develop a sense of belonging to the university and build connections within a culturally diverse environment. Additionally, it is unclear whether choir participation can reduce loneliness and support mental health and wellbeing during the transitional year.

### 1.3. The Current Study

The UQ Voices choir was established in January 2022 at the University of Queensland (UQ) to address the significant challenges faced by international students during the COVID-19 pandemic, including increased loneliness and mental health issues resulting from travel restrictions, online-only teaching, and social isolation. As international borders reopened and travel restrictions eased, the choir aimed to welcome international students back to campus, foster social connections, and build a supportive community through collaborative musical activities such as regular rehearsals, performances, and culturally enriching activities (Bos et al., 2024). Drawing on the social cure framework, this study evaluates the impact of participation in the UQ Voices choir on international students’ sense of belonging, loneliness, and wellbeing. It also aims to examine whether choir participation fosters a shared group identity and develops psychological resources such as self-esteem, sense of control, meaning and purpose, and mood improvement. Finally, this study assesses how choir group identity correlates with these psychosocial resources, students’ sense of belonging to the university, loneliness, and overall wellbeing. The research is designed to test the following four hypotheses:

**H1.**  *Compared to controls, international students who participate in the choir will report an enhanced sense of belonging at university over time*.

**H2.**  *Compared to controls, international students who participate in the choir will report reduced levels of loneliness over time*.

**H3.**  *Compared to controls, international students who participate in the choir will report improved wellbeing over time*.

**H4a.**  *Among choir participants, there will be significant pre-post increases in their choir group identity and reported experiences of psychosocial resources (e.g., self-esteem, sense of control, meaning and purpose, and mood improvement)*.

**H4b.**  *Among choir participants, students’ choir group identity will positively correlate with their reported psychosocial resources, sense of belonging to the university, reduced loneliness, and improved wellbeing*.

## 2. Materials and Methods

### 2.1. Study Design

This non-randomised controlled study had a mixed within and between groups design, with two conditions: choir members and controls (international students at the same university who were not in the choir) and two time points: baseline (pre-survey) and after 6–8 weeks (post-survey).

### 2.2. Participants and Recruitment

This study involved 53 international students enrolled in any course at UQ, including undergraduate, postgraduate, English bridging courses, and foundation programmes. The choir condition consisted of 31 members of the UQ Voices choir (*M*_age_ = 24.90 years, *SD* = 6.02, 71.0% female), while the control condition included 22 international students who did not participate in the choir (*M*_age_ = 24.18 years, *SD* = 3.78, 72.7% female). As part of demographic data collection, participants also reported their ethnicity, relationship status, living situation, and music experience (i.e., whether they played an instrument, sang in a choir, or listened to music in their native language). Between January 2022 and May 2024, choir members were recruited using various methods, such as presentations/performances during the orientation week, in-class announcements, and personal networks. Also, the choir was advertised through student social media sites, international student associations, and the choir’s own Instagram account. The control group was recruited via word-of-mouth through choir members, ensuring a manageable and balanced sample size.

A priori power analysis was conducted using G*Power 3.1 (Faul et al., 2007) to determine the required sample size for a mixed ANOVA with a medium effect size (*f* = 0.25), *α* = 0.05, and a desired power of 0.90. The analysis indicated that a total sample size of 46 participants would be sufficient to detect a medium effect size. The actual sample size in this study was 53 participants, which ensured adequate power to detect the hypothesised effects.

### 2.3. Measures

Loneliness. Loneliness was assessed using the eight-item Loneliness Scale (Hays & DiMatteo, 1987), adapted from the UCLA Loneliness Scale (Russell et al., 1980). An example of the items is, “I feel left out”. Participants rated their responses on a four-point Likert scale, ranging from 1 = Never to 4 = Always. Total scores range from 8 to 32, with higher scores indicating greater loneliness. One study of Taiwanese undergraduate students using the same loneliness scale reported a mean score of 17.12 (*SD* = 2.10) (Wu & Yao, 2008). Given that this score is closer to the lower end of the scale, it suggests relatively lower loneliness in that sample and serves as a benchmark for understanding scores in similar populations. The scale demonstrated good internal consistency in the current study (Cronbach’s *α_pre_* = 0.71 and *α_post_* = 0.76, respectively).

Sense of Belonging to the University. Sense of belonging was measured using a single item adapted from the single-item social identification scale (Postmes et al., 2013), a validated and reliable measure of social group identification. The original phrasing, “I identify with students at UQ”, was modified to “I feel a sense of belonging at UQ” to make it easy to understand for international students. This question was designed to capture how comfortable, accepted, and included students feel in the university environment. Participants rated their responses on a five-point Likert scale, ranging from 1 = Strongly disagree to 5 = Strongly agree. Higher scores indicate a stronger sense of belonging.

Wellbeing. Wellbeing was assessed using the Short Warwick–Edinburgh Mental Wellbeing Scale (SWEMWBS; Ng Fat et al., 2017). This scale includes seven positively worded items, such as “I’ve been feeling optimistic about the future”, designed to measure mental wellbeing. Participants rated their responses on a five-point Likert scale, ranging from 1 = None of the time to 5 = All of the time. Total scores are calculated by summing the item responses, with higher scores indicating greater wellbeing. A recent study of Australian university students found that international students in 2022 reported a mean wellbeing score of 22.70 (*SD* = 4.64) on this scale (Dingle et al., 2024). The SWEMWBS demonstrated good internal consistency in the current study (Cronbach’s *α_pre_* = 0.87 and *α_post_* = 0.90, respectively).

Identification with the Choir. Choir group identity was measured using the four-item social identity scale (Doosje et al., 1995). It was adapted to include statements relating to the choir (e.g., “I see myself as a member of the choir”) rated on a five-point Likert scale, ranging from 1 = Strongly disagree to 5 = Strongly agree. The items were averaged to yield a score from 1 to 5, with scores above 3 (the neutral point) indicating group identification. The choir group identity scale demonstrated good internal consistency in the current study (Cronbach’s *α_pre_* = 0.87 and *α_post_* = 0.87, respectively, for the choir group).

Perceived Psychosocial Resources. Four items assessed the extent to which choir participation is perceived to develop psychosocial resources, including self-esteem, sense of control, meaning and purpose, and mood improvement. These items were adapted from the seven items used in a music project (Kyprianides & Easterbrook, 2020) and were drawn from social identity theory research (Draper & Dingle, 2021; Greenaway et al., 2015). Specifically, they assess self-esteem (“Singing in the UQ Voices choir gives me self-esteem”), control (“Singing in the UQ Voices choir gives me a sense of control”), meaning and purpose (“Singing in the UQ Voices choir gives me meaning and purpose”), and mood improvement (“Singing in the UQ Voices choir improves my mood”). Participants rated each statement on a five-point Likert scale, ranging from 1 = Strongly Disagree to 5 = Strongly Agree, with higher scores indicating greater perceived resources. The four items were treated as separate measures in subsequent analyses.

### 2.4. Procedure

Participants in the choir group attended 120-min rehearsals each week over the semester. Rehearsals were facilitated by a community choir director who arranged the songs in three- or four-part harmony using lyric sheets and audio recordings for choir members to listen to and rehearse. Each session comprised an icebreaker, physical and vocal warm-ups, sectional practice, and full choir run-throughs. Repertoire was co-created: students proposed songs, discussed options during rehearsal breaks, and voted anonymously. Leadership was shared, as the director facilitated rehearsals while students took on roles such as arranging music, leading sectionals, and organising social and performance activities, allowing international members to feel a sense of ownership and involvement (Bos et al., 2024). The retention rate across the rehearsal period was 88.7%.

When joining the choir, participants were provided with a QR code with the link to access the baseline pre-survey, which included participant information and consent in an online form. Students who provided consent completed the pre-survey using their personal devices during choir breaks or at their own time after the choir practice. After 6–8 weeks, they completed the post-survey following the same process. Control group participants also completed the pre- and post-surveys online, following the same timeline as the choir group. All participants received an AUD 20 voucher upon completing each survey. Choir group members were debriefed about this study either in person during a choir session or through an online debriefing sheet, while control group participants received the debriefing sheet online.

### 2.5. Data Analysis

A 2 × 2 mixed factorial ANOVA was conducted to examine the effects of group (choir vs. control) and time (pre vs. post) on loneliness, sense of belonging to university, and wellbeing. Interaction effects were tested to determine whether changes over time differed between the choir and control groups. Main effects of group and main effects of time were reported where relevant. Prior to conducting the analysis, data were screened for missing values, and assumptions of normality and homogeneity of variance were evaluated. A missing data analysis revealed that less than 5% of data were missing across all variables. All participants and variables were adequately sampled, meeting the criteria for inclusion in subsequent analyses (Schlomer et al., 2010). Also, the Little’s MCAR test result was nonsignificant, *χ*^2^ (48) = 52.48, *p* = 0.305, suggesting the data were missing completely at random (Little, 1988). Normality was assessed using skewness, kurtosis, and the Shapiro–Wilk test. All assumptions were met for the analysis, except for a deviation from normality in the Shapiro–Wilk test for sense of belonging to the university. Homogeneity of variance was tested using Levene’s test, which indicated that the assumption of equal variances was satisfied for all dependent variables (*p* > 0.05). Given the robustness of the mixed-design ANOVA to minor deviations, analyses proceeded as planned.

### 2.6. Choir-Only Analyses

We conducted additional analyses on the choir group to evaluate pre- to post- changes in choir identity and psychosocial resources. The same data-screening procedures, such as missing data analysis and tests of normality, were applied to this subsample, and no substantial violations of assumptions or missing data patterns emerged. Therefore, paired-sample *t*-tests were used to assess within-group changes in choir identity and each psychosocial resource (i.e., self-esteem, sense of control, meaning and purpose, mood). Pearson’s correlations were then conducted to examine whether choir group identity was associated with reported psychosocial resources, sense of belonging to the university, loneliness, and wellbeing at post-survey.

## 3. Results

### 3.1. Descriptive Results

As shown in Table 1, the average age of participants was 24.60 years (*SD* = 5.18), with most in the 21–25 age group. Gender distribution was similar, with a majority identifying as female (71.0% choir, 72.7% control). Both groups had a large proportion of Southeast Asian participants, though the control group had more Northeast Asian participants. Most participants were single, with choir participants often living in shared student accommodation (29.0%) or share houses (25.8%), while control participants predominantly lived in share houses (40.9%). Music experience differed between groups, with more choir participants currently playing an instrument or singing in a choir (41.9% choir, 13.6% control) and reporting past music activity (87.1% choir, 59.1% control). Listening to music in their native language was common in both groups.

### 3.2. Descriptives and Between-Group Tests at Baseline

Table 2 presents the baseline pre-survey means and standard deviations for sense of belonging to the university, loneliness, and wellbeing in each group, as well as the results of independent-sample *t*-tests. In all three outcome variables, there were no significant differences between the choir group and the control group at the baseline pre-survey, indicating that the groups were comparable at the start of this study. Overall, baseline pre-survey scores for loneliness (*M_choir_* = 17.81, *SD_choir_* = 3.53; *M_control_* = 17.82, *SD_control_* = 3.08) were consistent with previously published norms among Taiwanese university students (*M* = 17.12, *SD* = 2.10) (Wu & Yao, 2008), suggesting that students in our sample experienced levels of loneliness similar to other university student samples. Likewise, wellbeing scores (*M_choir_* = 24.84, *SD_choir_* = 4.80; *M_control_* = 22.82, *SD_control_* = 4.20) were comparable to those reported by the 2022 cohort of international students in an Australian university (*M* = 22.70, *SD* = 4.64) (Dingle et al., 2024), indicating that participants generally fell within typical ranges on these measures. Given that participants’ baseline pre-survey scores on loneliness and wellbeing were near established averages, there might have been limited room for improvement over time, which we explore further in the subsequent analyses.

### 3.3. Main Analyses

#### 3.3.1. Effects of Choir Participation Compared to Control

**Figure 1:**
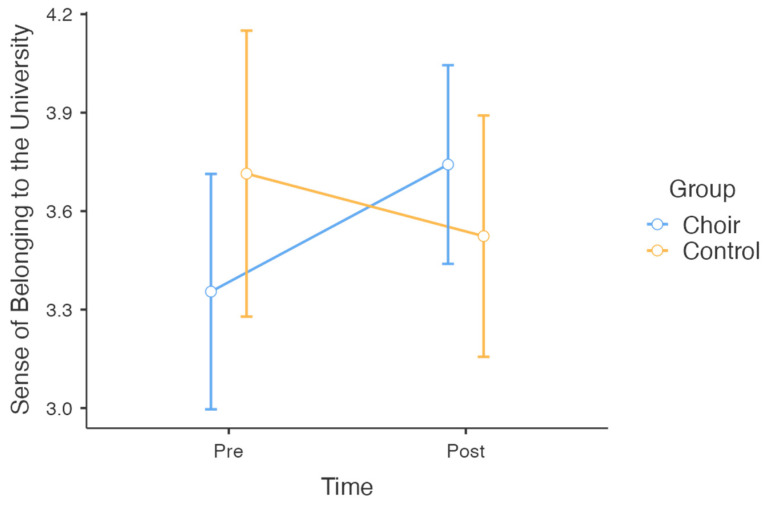
Changes in sense of belonging to the university across choir and control groups over time.

The results for the mixed factorial ANOVAs are shown in Table 3. The analysis for sense of belonging to the university showed a significant group × time interaction, *F*(1, 50) = 2.088, *p* = 0.021, *η*^2^*_p_* = 0.102, indicating that changes in the sense of belonging over time differed between the two groups (Figure 1). However, there were no significant main effects of group, *F*(1, 50) = 0.094, *p* = 0.760, *η*^2^*_p_* = 0.002, or time, *F*(1, 50) = 0.242, *p* = 0.420, *η*^2^*_p_* = 0.013. Follow-up *t*-tests showed that the choir group reported a significant increase in sense of belonging from pre (*M* = 3.35, *SD* = 1.02) to post (*M* = 3.74, *SD* = 0.97), *t*(30) = −2.26, *p* = 0.031, while the control group showed no significant change (*M* = 3.71, *SD* = 0.96 at pre; *M* = 3.46, *SD* = 0.67 at post), *t*(20) = 1.28, *p* = 0.214.

**Figure 2:**
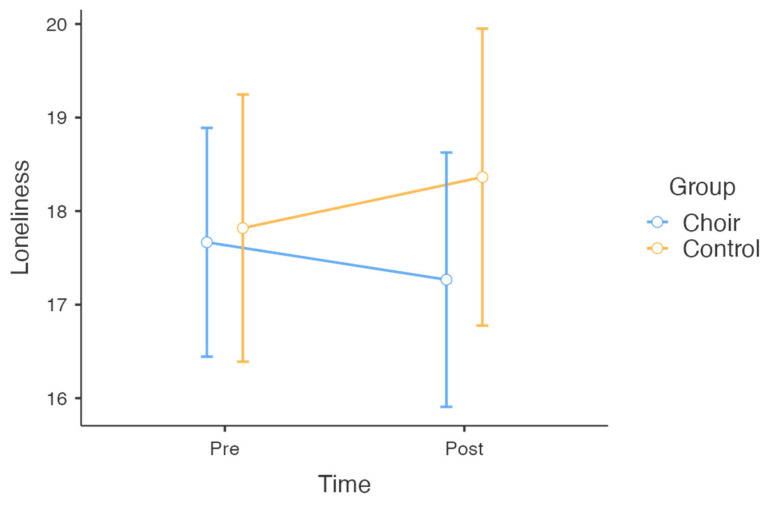
Changes in loneliness across choir and control groups over time.

For loneliness, there were no significant results for group × time interaction, *F*(1, 50) = 0.886, *p* = 0.351, *η*^2^*_p_* = 0.002 (Figure 2), indicating that changes in loneliness over time did not differ between the choir and control groups, and Hypothesis 2 was not supported. Additionally, there were no significant main effects of group, *F*(1, 50) = 0.536, *p* = 0.467, *η*^2^*_p_* = 0.011, or time, *F*(1, 50) = 0.021, *p* = 0.885, *η*^2^*_p_* = 0.000.

**Figure 3:**
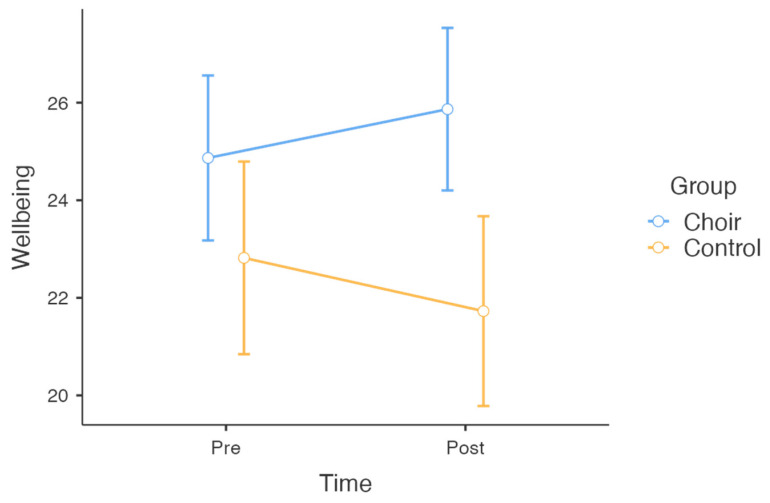
Changes in wellbeing across choir and control groups over time.

For wellbeing, the results showed a significant main effect of group, *F*(1, 50) = 7.69, *p* = 0.008, *η*^2^*_p_* = 0.133, suggesting that the choir group reported higher overall wellbeing compared to the control group across both time points (refer to Table 2). However, there was no significant main effect of time, *F*(1, 50) = 0.005, *p* = 0.943, *η*^2^*_p_* = 0.000, or group × time interaction, *F*(1, 50) = 2.69, *p* = 0.107, *η*^2^*_p_* = 0.051 (Figure 3).

#### 3.3.2. Pre–Post Changes in Choir Members’ Identity and Resources

Among choir members, there was a significant pre–post increase in choir identity, *t*(28) = −3.01, *p* = 0.006, Cohen’s *d* = −0.56, with mean scores increasing from *M* = 4.10 (*SD* = 0.64) at pre to *M* = 4.44 (*SD* = 0.62) at post (Table 4). Regarding psychosocial resources that students experienced through choir participation, self-esteem showed a significant increase, *t*(27) = −2.65, *p* = 0.013, Cohen’s *d* = −0.50. Similarly, meaning and purpose significantly increased from pre to post, *t*(26) = −2.53, *p* = 0.019, Cohen’s *d* = −0.49. However, no significant changes were observed for sense of control (*p* = 0.106) or mood improvement (*p* = 0.573).

#### 3.3.3. Correlations Between Choir Identity, Psychosocial Resources, and Outcomes

Correlations among choir identity, psychosocial resources, and key outcomes (sense of belonging, loneliness, and wellbeing) are presented in Table 5. Specifically, this study focuses on the correlation between choir identity and both psychosocial resources and key outcomes. Identification with the choir was positively and significantly correlated with self-esteem (*r* = 0.71, *p* < 0.001), sense of control (*r* = 0.47, *p* = 0.009), meaning and purpose (*r* = 0.56, *p* = 0.002), and mood improvement (*r* = 0.66, *p* < 0.001). This indicates that stronger identification with the choir was associated with greater psychosocial resources among choir members. Additionally, identification with the choir was significantly associated with a sense of belonging to the university (*r* = 0.43, *p* = 0.018) and wellbeing (*r* = 0.43, *p* = 0.018); however, no significant correlation was found between choir identification and loneliness (*r* = −0.26, *p* = 0.164).

## 4. Discussion

This study aimed to evaluate the impact of choir participation on international students’ sense of belonging, loneliness, and wellbeing. The findings provided a mix of support and challenges for these hypotheses. Choir participation significantly increased students’ sense of belonging and was associated with higher wellbeing, but it did not lead to a significant reduction in loneliness. Additionally, choir members reported higher overall wellbeing. Some psychosocial resources, such as self-esteem and meaning and purpose, increased over time, while others, like sense of control and mood improvement, showed no significant changes. These results highlight the potential benefits of choir participation in promoting social and psychological wellbeing among international students, which are explored further below.

One of the key findings of this study was that choir participation significantly increased students’ sense of belonging to the university, which supported Hypothesis 1. This finding aligned with previous research showing that choir singing encourages social connections and reduces isolation (Clift & Hancox, 2001; Gridley et al., 2011; Moss et al., 2018). However, this study highlights this benefit specifically in the context of international students, a group that has not been widely examined in previous choir-related research. This finding emphasises the potential of choirs as a culturally inclusive intervention to support social integration and community connection, addressing the challenges international students face in building meaningful relationships in a new environment.

From a theoretical perspective, these findings are consistent with social identity theory, which posits that shared group experiences promote a sense of belonging (Tajfel et al., 1979). In this context, participating in the UQ Voices choir, a university-based choir with members from the same institution, likely created a shared ‘choir identity’, where members felt part of the university choir group, further enhancing their sense of belonging to the university (Bos et al., 2024; Dingle et al., 2013, 2020; Draper & Dingle, 2021). Consistent with Hypothesis 4a, choir participants showed significant pre–post increases in their choir group identity and psychosocial resources, such as self-esteem and meaning and purpose (Cruwys et al., 2014; Greenaway et al., 2015; Jetten et al., 2015). These psychosocial resources likely provided the psychological foundation for students to feel more connected and integrated into the university environment. Additionally, in support of Hypothesis 4b, choir identification was positively associated with students reported psychosocial resources, including self-esteem, sense of control, meaning and purpose, and mood improvement. Furthermore, choir identification was significantly associated with their sense of belonging to the university and overall wellbeing. These findings extend the social cure framework (C. Haslam et al., 2018; Jetten et al., 2012) to a new age group and context by demonstrating how forming a new social identity through choir participation can promote belonging among university students. Moreover, this study builds on Cruwys et al.’s (2020) findings on social identity continuity by showing that forming a new group identity, such as through choir membership, is not only positive but also an effective strategy for fostering a sense of belonging and improving psychosocial outcomes during a significant life transition, such as starting university.

Unexpectedly, there were no significant changes in loneliness across groups or over time, failing to support Hypothesis 2. This contrasts with previous research suggesting that choir participation could reduce loneliness and social isolation while increasing social connections (Clift & Hancox, 2010; Gridley et al., 2011; Moss et al., 2018; Williams et al., 2018). One explanation for this non-significant result may be the ‘typical’ (i.e., not elevated) baseline pre-survey loneliness levels across groups (refer to Table 2), which were comparable to the published mean (*M* = 17.12, *SD* = 2.10) for Taiwanese students reported by Wu and Yao (2008), leaving limited room for choir participation to make a noticeable difference. Another possibility is that loneliness may be more effectively managed through close personal relationships, such as familial or romantic connections and long-term friendships (Bernardon et al., 2011). While choir participation can foster new social connections, these may not be sufficient to significantly impact loneliness if individuals already have strong existing relationships that provide emotional support. Additionally, while choir participation significantly enhanced students’ sense of belonging, it did not lead to a significant reduction in loneliness. This may reflect the distinct nature of these two constructs, as the sense of belonging to the university involves feelings of being accepted, valued, and connected to the university community (Glass & Westmont, 2014). Joining a choir can quickly provide students with the experience of social connection, inclusion, and group identity, which could explain a significant increase in their sense of belonging. Loneliness, however, is a deeper emotional experience of social disconnection or lack of fulfilling relationships (Yanguas et al., 2018). As such, participation in a choir may not immediately reduce the deeper emotional experience of loneliness, especially when influenced by long-standing factors such as cultural adjustment, academic stress, or limited social networks beyond the choir (Neto, 2021; Sawir et al., 2008).

In addition, Hypothesis 3 was partially supported, as a significant main effect of group was found, with students in the choir group reporting higher overall wellbeing than those in the control group. Importantly, there were no significant differences in wellbeing at baseline pre-survey between the two groups (refer to Table 2), and the significant difference observed at post-survey suggests a positive impact of choir participation. This finding aligns with previous research highlighting the role of group singing in mental health and wellbeing (Bailey, 2021; Clift & Hancox, 2001; Glew et al., 2021; Van Der Sandt & Nardi, 2024). While most previous studies have not focused specifically on international students, our findings suggest that the wellbeing benefits of choir participation can also extend to this population. However, the absence of a significant interaction effect indicates that these benefits did not vary over time within the study period, suggesting the need for further research to explore the long-term effects of choir participation on wellbeing.

The current study has a few limitations. First, the short duration of this study may not have been sufficient to capture long-term changes in constructs such as loneliness and wellbeing. Future studies could incorporate longer follow-up periods, as suggested by Dingle et al. (2019), with at least three assessment points (e.g., baseline pre-survey, post-intervention, and a 6-month follow-up) to better understand the sustained effects of choir participation. Second, the lack of random assignment to the choir and control groups introduces potential selection bias, as participants who chose to join the choir may differ from those in the control group in ways not measured by this study. While randomisation is generally preferred in group singing research (Dingle et al., 2019), it was not feasible in this context due to the voluntary nature of choir participation. Furthermore, no restrictions were placed on other activities that choir or control group members may have engaged in during this study. This lack of control could have introduced additional variability into the data, potentially reducing the specific effects attributable to choir participation. Future studies could address this limitation by employing strategies such as propensity score matching to statistically account for baseline differences or by using larger samples to better control for variability between groups. Also, this study was conducted at a single university, which may limit the applicability of the findings to other institutions or cultural contexts. Future studies could aim to include larger, more diverse samples across multiple universities and cultural settings to enhance the generalisability and applicability of the results.

## 5. Conclusions

This study highlights the significant potential of choir participation as an accessible intervention to enhance international students’ wellbeing and sense of belonging. These findings emphasise the value of group-based activities in creating social bonds and supportive networks for students adapting to new cultural and academic environments. Programmes like the UQ Voices choir bring together international students from diverse cultures and languages, offering opportunities to share unique backgrounds through group singing, fostering a group identity, and providing psychosocial resources such as self-esteem, a sense of control, and meaning and purpose. However, the sample was modest, and the intervention is short, so confirmation in larger cohorts with longer follow-up will be valuable. As higher education institutions become more globalised, creative initiatives like choirs could enrich the university experience and improve wellbeing across cultural contexts. Future research could investigate the mechanisms driving these benefits, such as shared identity, psychosocial resources, and emotional expression, as well as examine long-term and cross-cultural impacts.

## Figures and Tables

**Table 1:** Participant demographics of choir and control groups.

	Choir (*N* = 31)	Control (*N* = 22)
Age (*M* = 24.60, *SD* = 5.18)	*M* = 24.90, *SD* = 6.02	*M* = 24.18, *SD* = 3.78
18–20 years	9 (29.0%)	5 (22.7%)
21–25 years	12 (38.7%)	9 (40.9%)
26+ years	10 (32.3%)	8 (36.4%)
Gender		
Female	22 (71.0%)	16 (72.7%)
Male	6 (19.4%)	2 (9.1%)
Non-binary	1 (3.2%)	3 (13.6%)
Prefer not to say	1 (3.2%)	1 (4.5%)
Ethnicity		
Southeast Asian	14 (45.2%)	8 (36.3%)
Northeast Asian	5 (16.1%)	7 (31.8%)
South Central Asian	3 (12.9%)	1 (4.5%)
The Americas	2 (6.5%)	-
West European	2 (6.5%)	-
East European	1 (3.2%)	-
Multiple Ethnicities	-	1 (4.5%)
Aboriginal or Torres Strait Islander	-	1 (4.5%)
Other	4 (12.9%)	3 (9.7%)
Relationship Status		
Single	22 (71.0%)	14 (63.6%)
In a relationship (living together)	3 (9.7%)	4 (18.2%)
In a relationship (not living together)	4 (12.9%)	2 (9.1%)
Married	2 (6.5%)	1 (4.5%)
Separated/Divorced	-	1 (4.5%)
Living Situation		
In a shared student accommodation	9 (29.0%%)	3 (13.6%)
Living with family	6 (19.4%)	1 (4.5%)
Living alone	5 (16.1%)	3 (13.6%)
In a share house	8 (25.8%)	9 (40.9%)
In a studio student accommodation	3 (9.7%)	3 (13.6%)
In a UQ residential college	-	-
Music Experience		
Currently playing an instrument or singing in a choir (not UQ Voices)	13 (41.9%)	3 (13.6%)
Past experiences in playing an instrument or singing in a choir	27 (87.1%)	13 (59.1%)
Usually listening to music in native language	21 (67.7%)	15 (68.2%)

**Table 2:** Descriptive statistics and baseline pre-survey comparisons for dependent variables across groups and time.

	Choir (*N* = 31)		Control (*N* = 22)		Baseline Pre-Survey Comparison (*t*, *p*)
	Pre	Post	Pre	Post	
	Mean (*SD*)	Mean (*SD*)	Mean (*SD*)	Mean (*SD*)	
Sense of Belonging to the University (range: 1–5)	3.35 (1.02)	3.74 (0.97)	3.71 (0.96)	3.46 (0.67)	*t*(50) = −1.28, *p* = 0.207
Loneliness (range: 8–32)	17.81 (3.53)	17.27 (3.81)	17.82 (3.08)	18.36 (3.55)	*t*(51) = −0.013, *p* = 0.997
Wellbeing (range: 7–35)	24.84 (4.80)	25.87 (4.83)	22.82 (4.20)	21.73 (4.11)	*t*(51) = 1.59, *p* = 0.119

**Table 3:** Results of mixed factorial ANOVAs examining the impact of group and time on the dependent variables.

	Group Main Effect	Time Main Effect	Group × Time Interaction
Sense of Belonging to the University	*F*(1, 50) = 0.094, *p* = 0.760, *η*^2^*_p_* * = 0.002	*F*(1, 50) = 0.242, *p* = 0.420, *η*^2^*_p_* = 0.013	*F*(1, 50) = 2.088, *p* = 0.021, *η*^2^*_p_* = 0.102
Loneliness	*F*(1, 50) = 0.536, *p* = 0.467, *η*^2^*_p_* = 0.011	*F*(1, 50) = 0.021, *p* = 0.885, *η*^2^*_p_* = 0.000	*F*(1, 50) = 0.886, *p* = 0.351, *η*^2^*_p_* = 0.002
Wellbeing	*F*(1, 50) = 7.69, *p* = 0.008, *η*^2^*_p_* = 0.133	*F*(1, 50) = 0.005, *p* = 0.943, *η*^2^*_p_* = 0.000	*F*(1, 50) = 2.69, *p* = 0.107, *η*^2^*_p_* = 0.051

* *η*^2^*_p_* represents the effect size.

**Table 4:** Pre–post results for choir identity and psychosocial resources among choir members.

	Pre	Post	*t*	*df*	*p*	Cohen’s *d*
	Mean (*SD*)	Mean (*SD*)				
Identification with the UQ Voices Choir (range: 1–5)	4.10 (0.64)	4.44 (0.62)	−3.01	28	0.006	−0.56
Self-esteem (range: 1–5)	3.89 (0.74)	4.39 (0.74)	−2.65	27	0.013	−0.50
Sense of Control (range: 1–5)	3.64 (0.83)	4.00 (0.98)	−1.67	27	0.106	−0.32
Meaning and Purpose (range: 1–5)	3.81(0.85)	4.23 (0.86)	−2.53	26	0.019	−0.49
Mood Improvement (range: 1–5)	4.57 (0.57)	4.64 (0.56)	−0.57	27	0.573	−0.11

**Table 5:** Correlations between choir identity, psychosocial resources, and key outcomes in the choir group at post-survey.

	Variables	1	2	3	4	5	6	7	8
1.	Identification with the UQ Voices choir	—							
2.	Self-esteem	0.71 **	—						
3.	Sense of Control	0.47 **	0.73 **	—					
4.	Meaning and Purpose	0.56 **	0.78 **	0.57 **	—				
5.	Mood Improvement	0.66 **	0.60 **	0.54 **	0.62 **	—			
6.	Sense of Belonging to the University	0.43 *	0.28	0.34	0.28	0.23	—		
7.	Loneliness	−0.26	−0.09	−0.05	−0.14	−0.24	−0.42 *	—	
8.	Wellbeing	0.43 *	0.36 *	0.54 **	0.44 *	0.45 *	0.66 **	−0.60 **	—

* for *p* < 0.05 and ** for *p* < 0.001. This study specifically focuses on the correlation between identification with choir and psychosocial resources, sense of belonging to the university, loneliness, and wellbeing.

## Data Availability

The data that support the findings of this study are available from the corresponding author, R.H., upon reasonable request.
